# Novel ZAP-70-Related Immunodeficiency Presenting with Epstein–Barr Virus Lymphoproliferative Disorder and Hemophagocytic Lymphohistiocytosis

**DOI:** 10.1155/2021/6587323

**Published:** 2021-06-19

**Authors:** Moriah Forster, Timothy Moran, Anne Beaven, Timothy Voorhees

**Affiliations:** ^1^Department of Medicine University of North Carolina, 126 MacNider Hall, CB #7005 Chapel Hill, NC 27599, USA; ^2^Division of Immunology, Department of Pediatrics, University of North Carolina, 030 MacNider Hall, CB #7231 Chapel Hill, NC 27599, USA; ^3^Division of Hematology, Department of Internal Medicine, Lineberger Comprehensive Cancer Center, University of North Carolina, 170 Manning Drive, CB #7305 Chapel Hill, NC 27599, USA

## Abstract

Zeta-chain-associated protein kinase 70 (ZAP-70) plays an integral role in the T-cell antigenic receptor complex. A deficiency of this kinase leads to a phenotype of severe combined immunodeficiency, while hypomorphic mutations of the kinase lead to more mild immunodeficiency phenotypes. We present a case of a 21-year-old patient with lymphadenopathy who was found to have Epstein–Barr virus (EBV) lymphoproliferative disease (LPD) and the development of hemophagocytic lymphohistiocytosis (HLH). On further workup, the patient was ultimately found to have a homozygous intrionic mutation in ZAP-70. This is a novel ZAP-70 mutation (c.1623 + 5G > A) associated with combined immunodeficiency and an EBV-positive LPD. A primary immunodeficiency is important to consider in a young, otherwise healthy patient presenting with an EBV-positive LPD.

## 1. Introduction

Zeta-chain-associated protein kinase 70 (ZAP-70) is a cytoplasmic kinase which plays an integral role in the T-cell antigenic receptor complex, leading to T-cell receptor activation, T-cell development, immunity, and tolerance [1–7]. ZAP-70 deficiency typically presents with recurrent infections in the first months of life, low or absent CD8 positive T cells, normal to increased nonfunctional CD4-positive T cells, and defective B-cell antibody production—essentially severe combined immunodeficiency (SCID) [1–4, 6–8]. More mild immunodeficiency phenotypes have been described with hypomorphic genetic mutations leading to decreased ZAP-70 expression or function, as opposed to the absence of ZAP-70 expression [3, 7].

As ZAP-70 plays a role in the T-cell antigenic receptor complex, cases of ZAP-70 mutations presenting with dysregulation of Epstein–Barr virus (EBV) infection have been reported [1, 3, 4]. Defects in the lymphocytic cytotoxic pathway, T-cell signaling pathway, and the T- and B-cell interaction put patients at risk of EBV-related disease [9–13]. The inability to control EBV infection can lead to some patients developing EBV-positive B-cell lymphomas, chronic active EBV infections, and hemophagocytic lymphohistiocytosis (HLH) [9, 12–16]. We present a case of a 21-year-old patient with a not previously described mutation in ZAP-70 resulting in a combined immunodeficiency who presented with EBV-positive lymphoproliferative disorder as well as HLH.

## 2. Case Description

A 21–year-old man with a past medical history of frequent childhood ear and sinus infections presented with shortness of breath due to a left mainstem bronchus collapse. A Positron Emission Tomography and Computed Tomography (PET/CT) scan was obtained revealing metabolically active lymph nodes in the cervical, mediastinal, hilar, abdominal, and pelvic regions (max SUV 4.9) without an obvious cause of his lung collapse.

A right inguinal lymph node was biopsied which revealed paracortical expansion by a polymorphous atypical infiltrate composed of small- and medium-sized CD20-positive lymphocytes. EBER highlighted the cells corresponding to CD20-positive B cells. The high density and marked atypia of the EBV-positive cells did not favor infectious mononucleosis; however, the preserved nodal architecture also argued against EBV-positive diffuse large B-cell lymphoma. Ultimately, an EBV-positive lymphoproliferative disease (LPD) was favored without evidence of large cell transformation.

Six months after his initial PET/CT scan, he was referred to the University of North Carolina for evaluation and had yet to receive therapy for his LPD. At the time of this evaluation, he complained of intermittent fevers, abdominal pain, and headache. An EBV viral load was >1 million copies/ml which was elevated from where it had been previously in the 1,000 copies/ml range two and three months prior. A PET/CT scan revealed progressive lymphadenopathy and the development of splenomegaly (19.4 cm) with intense FDG avidity. Labs revealed a new anemia (Hgb 12.3 g/dL from previous baseline 17.0–17.9 g/dL two months prior), a new thrombocytopenia (127 × 10^9^/L from previous baseline 207–239 × 10^9^/L), mild transaminitis (AST 480 U/L, ALT 231 U/L elevated from previous normal values two months prior), hyperferritinemia (1170 ng/ml), and hypertriglyceridemia (252 mg/dL). He had significantly elevated soluble IL-2 receptor (26,320 pg/mL). NK cells from the peripheral blood were analyzed for NK-cell function using a chromium release method testing the ability to lyse target cells at four effector to target ratios. NK-cell function was determined to be normal. Without confirming the presence of hemophagocytosis, he met 6 of 8 HLH-2004 diagnostic criteria, consistent with a diagnosis of HLH.

Given the EBV viremia that had worsened over time and a diagnosis of EBV-positive LPD in an otherwise healthy 21-year old, an immune workup was pursued. He had no parental consanguinity. The absolute lymphocyte count was mildly low at 715 cells/uL. The absolute CD19 B-cell count was normal at 130 cells/uL as was the absolute CD16/56 NK-cell count at 227 cells/uL. T-cell subsets were not assessed clinically. He had an absolute CD4 count of 314 cells/uL and absolute CD3 count of 715 cells/uL. His immunoglobulins were low with IgM of 33 mg/dL and total IgG of 492 mg/dL. His IgA was normal at 99.3 mg/dL. IgG subclasses 1–4 were low at 266, 159, 11.8, and 1.9 mg/dL, respectively. HIV antigen and antibodies were negative. Mitogen studies showed significantly decreased proliferative responses to phytohemagglutinin (PHA) for both total CD45+ and CD3+ cells (7.3% and 14.3%, respectively, with normal ranges being ≥49.9% and ≥58.5%, respectively). Sequencing analysis from the peripheral blood with a 207 gene Invitae Primary Immunodeficiency Panel yielded a homozygous intronic mutation in *ZAP-70* (c.1623 + 5G > A) consistent with a variant of undetermined significance (VUS). He was also found to have two additional heterozygous VUS in the *CD79B* gene (c.152G > A) and *ORAI1* gene (c.141_142ins37). Finally, ZAP-70 flow cytometry showed normal ZAP-70 expression in T cells and NK cells.

**Figure 1:**
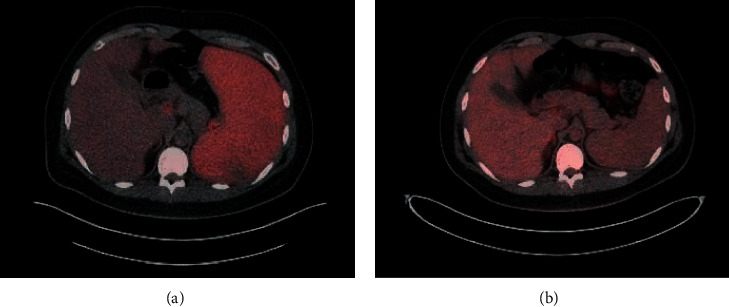
PET/CT comparison after 4 weekly dose of rituximab. Pretreatment PET/CT (a) showing FDG-avid splenomegaly. PET/CT after 4 weekly doses of rituximab 375 mg/m^2^ (b), demonstrating a complete response.

**Figure 2:**
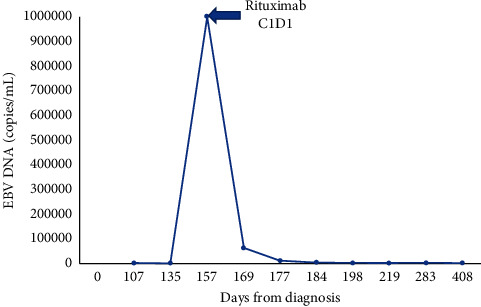
Epstein–Barr virus (EBV) viral loads over time. Day 0 is defined as the day of diagnosis with EBV-positive lymphoproliferative disorder. EBV DNA quantification from the peripheral blood at various time points before and after starting rituximab therapy. C1D1 = cycle 1, day 1 of rituximab. EBV quantification was undetectable after 60 days following the start of rituximab therapy.

The patient's treatment was approached similarly to a paradigm for LPDs in the post-organ-transplant setting. He received a risk-stratified approach with rituximab 375 mg/m^2^ weekly for four weeks followed by a PET/CT scan showing a complete response (Figure 1). He completed 4 additional doses of rituximab 375 mg/m^2^ consolidation every 3 weeks. In addition, he also received 4 doses of dexamethasone 40 mg daily given the presence of HLH and 4 weeks of a valganciclovir taper. His EBV viremia cleared four months after starting therapy, (Figure 2). He remains in a complete remission from his EBV lymphoproliferative disorder and HLH for over 14 months.

## 3. Discussion

The *ZAP-70* gene encodes a cytoplasmic tyrosine kinase, ZAP-70, that plays a role in T-cell receptor signaling [1–7]. Multiple different mutations in this gene lead to ZAP-70 deficiency which classically presents with CD8 lymphopenia and a SCID-like phenotype during infancy [1–8, 17]. Generally, the treatment requires hematopoietic stem cell transplant for survival past childhood [3, 7]. There are now case reports of patients with hypomorphic mutations presenting after infancy with EBV-positive LPDs, HLH, and autoimmunity with less severe immunodeficiency [1, 2, 4, 7]. Some of these mutations allow residual expression of the wild-type ZAP-70 kinase. We report a novel ZAP-70 mutation (c.1623 + 5G > A) associated with combined immunodeficiency and an EBV-positive LPD.

Given that ZAP-70 expression was preserved in T cells and NK cells in our patient, we suspect that this intronic mutation may affect RNA splicing and ultimately ZAP-70 function. His older age and lack of severe childhood illnesses are consistent with a hypomorphic mutation. Furthermore, his mitogen studies support the hypothesis of altered ZAP-70 function. Unfortunately, RNA splicing analyses were unable to be performed given that RNA isolation was not obtained from the patient during the time of his clinical care.

Of particular interest in this case was the patient's remarkable response to rituximab monotherapy in the setting of uncontrolled EBV viremia and a rapid evolution of his EBV-positive LPD. With an apparent underlying cellular and humoral immunodeficiency accompanying the diagnosis, we were hesitant to commit to multiagent chemotherapy. We favored the risk-stratified approach of induction rituximab followed by rituximab consolidation, previously reported in patients after solid organ transplant who have iatrogenic cellular and humoral immunodeficiencies due to chronic immunosuppression [18]. While this approach has been well described in the posttransplant lymphoproliferative setting, little data have been reported for EBV-positive LPDs due to combined immunodeficiencies [9, 12, 14, 16, 19–21].

## 4. Conclusions

In conclusion, a primary immunodeficiency is important to consider in a young, otherwise healthy patient presenting with an EBV-positive lymphoproliferative disorder. We report a new ZAP-70 mutation (c.1623 + 5G > A) which appears to be associated with a late-onset combined immunodeficiency and warrants further study. Finally, the risk-stratified, rituximab monotherapy approach should be a consideration for immunocompromised patients with EBV-positive LPDs, even in the absence of prior organ transplant.
